# Service-Specific Surveying to Support Person-Centered HCBS for Older Adults and Their Caregivers

**DOI:** 10.1093/geroni/igaa057.247

**Published:** 2020-12-16

**Authors:** Caitlyn Walsh, Jessica Tice

**Affiliations:** Florida Department of Elder Affairs, Tallahassee, Florida, United States

## Abstract

The Florida Department of Elder Affairs (DOEA) annually surveys clients receiving state funded home and community-based services (HCBS) to measure their satisfaction with services. Historically, the same survey instrument was used each year, to afford question-level comparisons across time. However, in 2015 internal contradictions were identified between individual-level satisfaction ratings and qualitative statements made by the respondents later in the survey. The high rates of satisfaction typical in survey responses were also contradicted by findings from a comprehensive program evaluation which revealed high percentages of clients who terminated their services and many caregivers reporting strain and varying types of personal crisis. To address these issues, the annual Client Satisfaction Survey and methodology was redesigned to be more specific regarding details about the delivery of direct services, and the sampling methodology was revised to constrain to the recipients of discreet service types. The results from these new service-level surveys will be presented for each of three direct services: case management, personal care, and homemaker. Findings revealed differences across regions in the state, and highlighted the frustration experienced by HCBS clients with high worker turnover and low training for special conditions, such as Alzheimer’s or related dementia. Complaints and suggestions collected from clients and caregivers were shared with program managers for consideration in changes to policies, training, and other areas of service improvement toward becoming more person-centered. Overall, this service-oriented approach to surveying has yielded more actionable results and has been adopted by DOEA as the preferred method for all client-level surveying.

